# Nation-Based Occurrence and Endogenous Biological Reduction of Mycotoxins in Medicinal Herbs and Spices

**DOI:** 10.3390/toxins7104111

**Published:** 2015-10-14

**Authors:** Kee Hun Do, Tae Jin An, Sang-Keun Oh, Yuseok Moon

**Affiliations:** 1 Laboratory of Mucosal Exposome and Biomodulation, Department of Biomedical Sciences, Pusan National University School of Medicine, Yangsan 50612, Korea; E-Mail: tkh3515@hanmail.net; 2 Department of Herbal Crop Research, National Institute of Horticultural & Herbal Science, RDA, Eumseong 55365, Korea; E-Mail: atj0083@korea.kr; 3 Department of Applied Biology, College of Agricultural & Life Sciences, Chungnam National University, Daejeon 34134, Korea; E-Mail: sangkeun@cnu.ac.kr; 4 Research Institute for Basic Sciences and Medical Research Institute, Pusan National University, Busan 46241, Korea

**Keywords:** mycotoxins, herbal medicine, spice, aflatoxin, ochratoxin A, fumonisin

## Abstract

Medicinal herbs have been increasingly used for therapeutic purposes against a diverse range of human diseases worldwide. Moreover, the health benefits of spices have been extensively recognized in recent studies. However, inevitable contaminants, including mycotoxins, in medicinal herbs and spices can cause serious problems for humans in spite of their health benefits. Along with the different nation-based occurrences of mycotoxins, the ultimate exposure and toxicities can be diversely influenced by the endogenous food components in different commodities of the medicinal herbs and spices. The phytochemicals in these food stuffs can influence mold growth, mycotoxin production and biological action of the mycotoxins in exposed crops, as well as in animal and human bodies. The present review focuses on the occurrence of mycotoxins in medicinal herbs and spices and the biological interaction between mold, mycotoxin and herbal components. These networks will provide insights into the methods of mycotoxin reduction and toxicological risk assessment of mycotoxin-contaminated medicinal food components in the environment and biological organisms.

## 1. Introduction

Herbal medicine has been increasingly used for therapeutic purposes against a diverse range of human diseases worldwide. However, contaminated chemicals in herbal medicines produce severe problems [1,2] that have seriously affected the value of herbal products and damaged human health. As one of the major contaminants, mycotoxins are the secondary metabolites produced by various species of fungi, such as *Aspergillus*, *Alternaria*, *Penicillium* and *Fusarium* [3], and trigger several ailments of the kidneys, liver, digestive tract, skin, respiratory organs, genital organs and nervous system [4,5]. More than 400 types of mycotoxins have been identified in the world to date. Among mycotoxins, aflatoxins (AFs), ochratoxin A (OTA), fumonisins (FBs), zearalenone (ZEA) and deoxynivalenol (DON) are the most frequently detected mycotoxins in herbal medicines. Generally, contamination can occur either in the pre-harvest or in the post-harvest and storage stages. Climate change, poor storage and damage from insects or harvest processing make them more susceptible to mycotoxin contamination [6]. The present review addresses the occurrence of mycotoxins in medicinal herbs and spices and the biological interaction between mold, mycotoxin and herbal components to get better exposure and toxicity assessments of the mycotoxin mixed with the health-promoting natural components.

## 2. The Global Occurrence of Mycotoxins in Medicinal Herbs and Spices

Information on mycotoxin occurrence in medicinal herbs and spices from different areas was compared based on the literature (Table 1). Different environmental conditions, agronomic practices and post-harvest processes, including storage and drying, resulted in a wide spectrum of mycotoxin contamination levels in medicinal herbs and spices.

Among Indian herbal samples, black pepper and long pepper are the most highly contaminated with AFB_1_. Out of the 150 samples, 43% were contaminated with AFB_1_, 6% with OTA, 6% with citrinin and 4% with ZEA. Crude samples of all 12 medicinal plants and spices [7] were randomly collected from gunny bags, metal containers, glass containers, wooden boxes and the bare ground in different store houses in India. Especially, samples collected from bags and the bare ground showed a significantly higher occurrence of mycotoxins than those from metal containers, glass containers and wooden boxes, suggesting an association of the storage conditions with mycotoxin production. In another report, 17 out of 84 samples [8] of medicinal herbs and spices from India were found to be contaminated with AFB_1_ and OTA. No mycotoxins were found in herbal samples of cinnamon, saffron, curcuma, rose or lesser galangal. All of the 84 medicinal herbs obtained from India were free of penicillic acid, ZEA and T-2 toxin [8]. Since mycotoxin quantitation in both reports was based on relatively old methods, such as thin layer chromatography and its subsequent UV or fluorescence spectrometric detection, more sensitive analytical methods to detect lower levels of mycotoxin contamination are also required to get the international recognition using the official methods of analysis from Association of Official Analytical Chemists (AOAC) international.

Mycotoxin occurrence in traditional Chinese herbal medicines has been extensively investigated. Different from the Indian surveys, the total of 51 dried samples of traditional Chinese medicinal herbs were examined for the mycotoxin contamination in 2010 using ultra-high-performance liquid chromatography-tandem mass spectrometry (UHPLC-MS/MS) [9]. The more accurate and sensitive analyses demonstrated that only four samples were found to have low levels of OTA and OTB contamination. Liu *et al.*, found that 27 out of 174 total samples of Chinese herbs were contaminated with aflatoxins, which indicates a 15.5% incidence rate of aflatoxin contamination. Products from Guangxi province showed the highest levels of contamination, with aflatoxins at up to 290.8 μg/kg in 27 contaminated samples. The mycotoxin analysis in Chinese herbal products was also performed using the immunoaffinity pre-column and HPLC-MS/MS [10]. Despite the relatively low incidence rate of aflatoxin contamination in Chinese herbs, the high levels of AFs in some medicinal herbs were high enough to cause serious health problems [10].

In Africa, 16 samples of traditional medicinal herbs from South Africa revealed the presence of Fb_1_ in 13 samples at up to 139 μg/kg. However, all of the 16 samples from South Africa using HPLC and MS analysis were free of AFB_1_ contamination [11]. In Morocco, spices, such as pepper, paprika, cumin, ginger and saffron, are extensively used in flavoring foods and medications. Spices are usually dried on the ground in an open space. These are unsuitable conditions in regions with a tropical climate, which poses a high risk of fungal contamination and is favorable for mycotoxin production. Among the total of 55 samples of four types of spices, 14 samples of red paprika contained higher levels of aflatoxins (9.68 μg/kg), a 100% incidence rate [12].

In Saudi Arabia, 50 different samples of 10 types of spices were analyzed for naturally-occurring mycotoxins using the thin layer chromatographic technique. A total of 10 different samples of five types of spices, such as anise, black cumin, black pepper, peppermint and marjoram, were found to be contaminated with aflatoxins at up to 40 μg/kg. Cardamom, cloves and ginger were free from aflatoxins and sterigmatocystin, whereas some spice samples of red pepper, cumin and marjoram were contaminated with sterigmatocystin at up to 25 μg/kg. This indicates that mycotoxin contamination of the spices from Saudi Arabia is of low incidence and occurs at only low concentrations. However, chronic exposure to even low levels of mycotoxins can be problematic, depending on the exposure population, because anise and cumin are commonly used as a carminative and expectorant for colic and flatulence in children.

In Turkey, aflatoxin contamination of dried figs has been reported [13,14,15]. As a result of reverse phase HPLC analysis, a total of 219 samples of dried figs demonstrated high incidence rates (47.5%) of aflatoxin at up to 278.04 μg/kg, whereas 2461 samples for export had a relatively lower incidence of aflatoxin contamination (23.6%) [15]. In addition, 75% of the 115 dried fig samples were found to be contaminated with extremely high levels of FB_1_, up to 3649 μg/kg [14].

**Table 1:** Occurrence of mycotoxins in medicinal herbs and spices.

Country of Origin	Sample Name	Type of Mycotoxin	Maximum Concentration of Mycotoxin (μg/kg)	Reference
India	Asparagus racemosus	AFB_1_	220	[7]
		AFB_2_	50	
		ZEA	100	
	Celery	AFB_1_	200	
		ZEA	70	
	Cinnamomum zeylanicum	AFB_1_	140	
	Cuminum cyminum	AFB_1_	310	
		ZEA	100	
	Elettaria cardamomum	AFB_1_	400	
		AFB_2_	210	
		OTA	50	
		ZEA	20	
	Emblica officinalis	AFB_1_	380	
		AFB_2_	80	
		AFG_1_	170	
		OTA	120	
		ZEA	190	
	Mesua ferrea	AFB_1_	270	
		AFB_2_	100	
	Long pepper	AFB_1_	570	
		AFB_2_	160	
		AFG_1_	190	
		OTA	80	
		ZEA	50	
	Black pepper	AFB_1_	510	
		AFB_2_	150	
		OTA	200	
		ZEA	100	
	Indica	AFB_1_	310	
	Baccala	AFB_1_	190	
	Ginger	AFB_1_	370	
		AFB_2_	220	
		AFG_1_	100	
		ZEA	70	
	Black cumin	AFB_1_	30	[8]
		OTA	35	
	Fennel	AFB_1_	160	
		OTA	80	
	Lime tree	AFB_1_	75	
	Wormwood	AFB_1_	25	
		OTA	20	
	Cinnamon	-	-	
	Peppermint	AFB_1_	25	
	Carob tree	AFB_1_	10	
	Chamomile	AFB_1_	145	
	Saffron	-	-	
	Curcuma longa	-	-	
	Worm wood	AFB_1_	90	
China	Rhizoma coptidis-1	OTA	0.4	[9]
	Rhubarb	OTA	0.2	
	Ephedra	OTA	0.3	
		OTB	0.4	
	Fructus mume	OTA	1.5	
		OTB	0.8	
	Baohe pills	DON	50.5	[16]
	Sping Jujuba seed	AFB_1_	4.67	[10]
		AFB_2_	0.89	
		AFG_1_	2.14	
	Barley	AFB_1_	1.72	
		AFB_2_	0.95	
	Areca seeds	AFB_1_	32.03	
		AFB_2_	2.73	
		AFG_1_	15.89	
	Biota seed	AFB_1_	25.33	
		AFB_2_	7.71	
		AFG_1_	0.59	
		AFG_2_	0.21	
	Cassia seed	AFB_1_	5.69	
		AFB_2_	1.81	
	Nutmeg	AFB_1_	239.62	
		AFB_2_	13.5	
		AFG_1_	34.21	
		AFG_2_	3.5	
	Bitter orange	AFB_1_	0.15	
		AFB_2_	0.77	
	Pharbitis seed	AFB_1_	0.47	
	Bitter apricot seed	AFB_1_	0.14	
		AFB_2_	0.07	
		AFG_1_	0.08	
		AFG_2_	0.09	
	White Aractylodes rhizome	AFB_1_	0.47	
		AFB_2_	0.06	
	Groomwell root	AFB_1_	1.03	
		AFB_2_	0.48	
	Japanese knotweed rhizome	AFB_1_	0.77	
		AFB_2_	0.32	
	Aractylodes rhizome	AFB_1_	0.58	
		AFB_2_	0.93	
	Corydalis rhizome	AFB_1_	68.4	
		AFB_2_	1.71	
		AFG_1_	0.95	
	Coix seeds	AFB_1_	0.09	
		AFB_2_	0.05	
		ZEA	211.4	[17]
South Africa	Uthuvana	FB_1_	40	[11]
	Isica Katha	FB_1_	87	
	Umsila Wengwe	FB_1_	117	
	Sibindi	FB_1_	30	
	Mudhora	FB_1_	25	
	Matunga	FB_1_	139	
	Mredeni	FB_1_	21	
	Red carrot	FB_1_	30	
	Roselina	FB_1_	126	
	Seloka	FB_1_	67	
	Thepe	FB_1_	26	
Saudi Arabia	Anise	AFB_1_, AFB_2_	38	[18]
	Black cumin	AFB_1_, AFB_2_	35	
	Black pepper	ST	40	
	Red pepper	AFB_1_, AFB_2_	25	
	Peppermint	AFB_1_, AFB_2_	17	
	Cumin	ST	20	
	Marjoram	AFB_1_, AFB_2_	12	
	Cinnamon	AFs	4.67	[19]
Morocco	Pepper	AFs	0.55	[20]
	Cumin	AFs	0.18	
	Ginger	AFs	9.10	
	Red paprika	AFs	9.68	
USA	Ginger	AFs	31	[21]
	Ginseng products	AFs	0.1	
		OTA	10	
	Ginseng root	AFs	16	[22]
	Kava-kava	AFB_1_	0.5	[23]
	Milk thistle	AFs	2.0	[24]
Spain	Sage leaves	AFs	25.2	[25]
		OTA	17.3	
		FBs	133.3	
		DON	102.2	
		Citrinin	273.2	
	Chamomile flower	AFs	161	
		FBs	90.0	
		ZEA	12.5	
		DON	191.5	
		Citrinin	51.6	
	Valerian root	AFs	15.8	
		FBs	96.7	
		T2	13.3	
		DON	64.7	
		Citrinin	20.5	
	Senna leaves	AFs	434.3	
		FBs	86.7	
		DON	35.2	
		Citrinin	68.6	
	Rhubarb	AFs	71.2	
		OTA	13.9	
		ZEA	24.4	
		T2	23.0	
		DON	58.4	
		Citrinin	42.9	
	Artichoke	AFs	12.1	
		T2	29.8	
		DON	200.2	
		Citrinin	29.8	
	Boldus	AFs	86.6	
		ZEA	10.3	
		T2	26.7	
		DON	343.5	
		Citrinin	25.8	
	Burdock root	AFs	10.3	
		ZEA	10.9	
		Citrinin	25.8	
	Dandelion	AFs	21.7	
		OTA	10.6	
		ZEA	17.0	
		DON	66.5	
		Citrinin	96.0	
	Frangula	AFs	64.7	
		ZEA	44.1	
		T2	12.6	
		DON	60.9	
		Citrinin	38.4	
	Ginkgo	AFs	23.3	
		T2	29.4	
		DON	134	
		Citrinin	354.8	
	Lemon verbena	AFs	37.7	
		ZEA	14.0	
		T2	28.6	
		DON	143.7	
		Citrinin	79.1	
	Olive leaves	AFs	77.6	
		ZEA	42.7	
		DON	149.9	
		Citrinin	14.9	
	Red tea	AFs	853.4	
		ZEA	11.2	
		T2	42.8	
		DON	179.9	
		Citrinin	22.3	
	Ribgrass	AFs	16.1	
		T2	256.9	
	Spearmint	AFs	29.7	
		DON	91.1	
		Citrinin	43.3	
	St Mary’s thistle	AFs	11.5	
		FBs	236.7	
		T2	35.6	
	Star anise	AFs	104.2	
		FBs	146.7	
		ZEA	10.1	
		T2	60.5	
		DON	321.2	
	Vervain	AFs	104.5	
		T2	20.4	
		DON	60.0	
		Citrinin	31.2	
	White tea	AFs	254.0	
		ZEA	11.2	
		T2	42.8	
		DON	259.1	
		Citrinin	19.7	
	Red paprika	OTA	73.8	[26]
	Licorice	OTA	252.8	[27]
Turkey	Chamomile	AFB_1_	38.9	[15]
	Rose hip	AFB_1_	52.5	
	Dried figs	AFs	278.04	[28]
		OTA	15.31	[29]
		FB_1_	3649	[14]

AF, aflatoxin; DON, deoxynivalenol; OTA, ochratoxin; T2, T2 toxin; FB, fumonisin; ZEA, zearalenone.

In Spain, 84 different samples of 42 types of medicinal and aromatic herbs were analyzed for multiple mycotoxins, including AF, OTA, ZEA, FBs, DON, T-2 toxin and citrinin, by using liquid chromatography with fluorescence detection (HPLC-FD) and HPLC-MS analysis. One hundred percent of the herbal samples were multi-contaminated with several mycotoxins, and 87% of samples were contaminated with four or more types of mycotoxins. Furthermore, 99% of the 84 samples were contaminated with T2, 98% with ZEA, 96% with AFs, 63% with OTA, 62% with DON, 61% with citrinin and 13% with FB_1_. Hierro *et al.* [26] analyzed five mycotoxins in 21 different samples of red paprika. Although 90% of the samples were contaminated with AFB_1_, the maximum levels of AFs were relatively low (AFB_1_: 3.8 μg/kg, AFB_2_: 0.7 μg/kg, AFG_1_: 1.1 μg/kg, AFG_2_:0.8 μg/kg). However, OTA contamination was found in 15 samples, and the maximum levels of OTA were relatively high (73.8 μg/kg).

Finally, in the USA, botanicals used for medicinal and health-promoting purposes, including ginseng, ginger and kava-kava, were assessed for aflatoxin contamination by using HPLC-based AOAC methods. In a recent study, relatively high levels of aflatoxins, over the national regulatory limits, were found in ginger products (31 μg/kg) [21]. In particular, ginger and ginseng root samples possessed more aflatoxins than other herbal products. In another report, 19% of 83 milk thistle samples were also found to be contaminated with aflatoxins ranging from 0.04 to 2.0 μg/kg [24].

Since most of the cited assessments were dependent on market-based sampling, other exogenous factors, such as the field environment, agronomic and the postharvest procedures, were not critically considered. Therefore, it is very hard to make direct comparisons of mycotoxin levels among countries. The procedure-based sampling and mycotoxin measurement would provide critical control points for the preventive reduction of fungal growth and mycotoxin production in the agricultural commodities. A recent study on the mycotoxin reduction in the medicinal plant adlay (Job’s Tears) demonstrated that the critical control point-based assessment was efficient to reduce the fungal growth and trichothecene production in the final product of the herbal medicine [30]. In addition to the diversities in the exogenous compounding factors affecting mycotoxin production in the medical herbs and spices, the quantitation methods should be the internationally recognized official methods of analysis, such as those by AOAC international.

## 3. Regulation of Fungal Growth and Mycotoxin Production by Components from Medicinal Herbs and Spices

Although the occurrence and exposure of mycotoxins in most medicinal herbs and spices in various countries are inevitable, many of the bioactive components in the medicinal herbs and spices are capable of regulating the fungal growth, mycotoxin production and their toxic actions in the exposed individuals. Therefore, the ultimate effects of mycotoxins on the exposure and toxicity can be potently influenced by the presence of endogenous antagonistic components in the edible matrix of the medicinal herbs and spices. Despite the efficiency of synthetic chemical compounds in eliminating mycotoxin-producing fungi and mycotoxin reduction, the residues of many chemicals pose health risks to humans and animals. Due to the toxicity of these exogenous xenobiotics used to reduce mycotoxin production and fungal growth, numerous studies have been conducted to identify effective natural product alternatives, such as herbs and spices [31,32]. Hussain *et al.* [33] studied the effect of herbal compounds and spices on toxin-producing *Aspergillus flavus* and *Aspergillus parasiticus*. Among nine samples of different herbal compound and spices, clove and clove oil showed complete growth-inhibitory activities against *A. flavus* and *A. parasiticus*, while ajwain, kalonji oil and turmeric produced only partial inhibition*.* Similarly, Azzouz *et al.* [34] examined the effects of spices on several toxigenic species of *Aspergillus* and *Penicillium*. Certain concentrations of cloves and cinnamon (more than 8%) completely inhibited fungal growth and their mycotoxin production. Mostafa *et al.* [35] assessed the inhibitory effects of 24 commercial spices and found that Chinese cassia, cinnamon, clove and thyme completely inhibited toxin production of four toxigenic *Aspergillus (A. flavus* and *A. versicolor)* and *Penicillium (P. citrinum* and *P. corylophilum)* species. Several reports have shown that powdered black and white pepper and cardamom inhibit aflatoxin production of different strains of *A. flavus* and *A. parasiticus* [36,37,38]. Atanda *et al.* reported that sweet basil leaves have a fungistatic effect on *A*. *parasiticus* CFR 223 and subsequent aflatoxin production, which suggests the possibility of their use against *Aspergillus* contamination of agricultural products [39]. In addition, essential oil extracted from medicinal herbs including cinnamon, marigold, spearmint, basil and quyssum, sufficiently inhibits the growth of fungi, such as *Aspergillus flavus*, *A. parasiticus*, *A. ochraceus* and *F. moniliforme* [40]. Moreover, Montes-Belmont and Carvajall *et al.* [41] and Basilico and Basilico *et al.* [42] demonstrated the antifungal activity of thyme, spearmint and basil on the toxigenic fungi *A. flavus*, *A. parasiticus*, *A. ochraceus*, *A. fumigatus* and *Fusarium* spp. These antifungal effects could be linked to common components known to have biological functions, such as α-pinene and β-pinene in basil, thyme and spearmint. Furthermore, basil oil contains ocimene and methyl chavicol as the most prevalent components. The major substances found in thyme oil are thymol and p-cymene. Many previous reports had demonstrated that cinnamon extract has effective antifungal activities against diverse toxigenic fungi. The three major components of cinnamon extracts (cinnamic aldehyde [43], *o*-methoxycinnamaldehyde [44] and carfone [45]) have proven anti-fungal activity. These anti-fungal plant-derived compounds have protective effects on the crop health by reducing detrimental fungal growth and mycotoxin actions, all of which are also beneficial for plant consumers, including human beings and domestic animals.

## 4. Regulation of Mammalian Toxicity of Mycotoxins by Components from Medicinal Herbs and Spices

If humans and animals are exposed to medicinal herbs and spices contaminated with mycotoxins, the ultimate biological sequelae would result from the interaction between the mycotoxins and components of the herbs and spices. Several medicinal herbs and spices are reported to be useful detoxification or protection agents against mycotoxin absorption, metabolism, distribution, excretion or toxicity. For instance, the induction of general metabolic enzymes in the liver, such as gamma-glutamyl transferase (γGT), aspartate aminotransferase (AST) and alkaline phosphatase (ALP), in response to aflatoxin exposure was significantly normalized by thyme oil treatment. Moreover, thyme oil enhances the excretion of aflatoxins and their metabolites via urine [46]. Several protective roles of sulforaphane have been identified by previous studies. In addition, Nayak *et al.* [47] observed that curcumin supplements, a bioactive principle of turmeric (*Curcuma longa* Linn), normalized aflatoxin-altered activities of lactate dehydrogenase and alanine transaminase (ALT).

In particular, aflatoxin-triggered oxidative stresses are also modulated by other diverse natural components in the medical herbs and spices. Similarly, the weight loss of chicks due to AFB_1_ was significantly restored by the supplementation with a turmeric (*C. longa*) powder containing curcumin [48]. In addition, turmeric powder alleviated AFB_1_-suppressed antioxidant activity, such as peroxides’ and superoxide dismutase (SOD) activity, and total antioxidant concentration in liver homogenates [48]. Akcam *et al.* [49] demonstrated that the intraperitoneal injection of caffeic acid phenethyl ester, an active component of honeybee propolis extract, exerts a protective effect against AFB_1_-induced hepatotoxicity. AFB_1_-altered levels of serum γGT, ALP, glutathione S-transferase (GST) and nitric oxide were significantly decreased by caffeic acid phenethyl ester in rats. The hepatoprotective roles of chitosan, derived from chitin found in crustacean shells, including *Pandalus borealis*, has been documented in several reports. Mosaad *et al.* [50] showed that chitosan nanoparticles ameliorate hepatotoxicity in response to AFB_1_ in the rat liver. Moreover, Subhapradha *et al.* [51] reported that β-chitosan sufficiently normalized oxidative stress-induced plasma AST and ALT levels in rats. The hepatoprotective roles of chitosan are most likely due to the elimination or prevention of free radicals by its antioxidant property. Cyanidin, a natural anthocyanidin found in diverse medicinal herbs as well as fruits and vegetables, such as grapes, bilberry, blackberry, cherry, cranberry, hawthorn, loganberry, acai berry, raspberry, red cabbage and red onion, has been reported to have a protective effect against AFB and OTA toxicity [52,53,54]. Guerra *et al.* [55] observed that cyanidin reduces AFB_1_ and OTA-mediated production of reactive oxygen species, suppression of protein and DNA synthesis and apoptosis in both hepatocytes and enterocytes. Moreover, carotenoid lycopene protects against the DNA damage, hepatotoxicity and renal oxidative stress caused by mycotoxins, including OTA and AFB_1_ [56,57,58]. Cyanidin and lycopene can be thus an efficient beneficial component to attenuate the mixture toxicity of the two genotoxic mycotoxins.

Moreover, Aboobaker *et al.* [59] examined the effect of various plant-derived phenolic compounds, including flavonoids, such as fisetin, kaempferol, morin, naringin and catechin, phenolic acids, such as caffeic acid and chlorogenic acid, and other phenolics, such as eugenol and vanillin, and found that they attenuate the hepatotoxicity of AFB_1_. Likewise, many types of herbs with potential uses for hepatotoxicity have been identified by several studies. Singh *et al.* [60] presented a list of 23 types of medicinal herbs and their active components with a hepatoprotective activity. Similar to the protective roles of chitosan or thyme on aflatoxin-mediated hepatotoxicity, these herbs also possess a hepatoprotective effect against mycotoxins accumulated in the liver (Table 2).

The suppressed oxidative stress was also associated with attenuated DNA damages and carcinogenesis. According to the results of Sheen (2001) [61], diallyl sulfide, an active principle of garlic, protects hepatocytes from AFB_1_-induced DNA damage by activating GST and glutathione peroxidase. Similarly, genistein, a phytoestrogen derived from soy beans and medicinal herbs, including *Flemingia vestita* and *Flemingia macrophylla*, showed an antigenotoxic effect against AFB_1_-caused mutagenesis [62]. In addition, sulforaphane derived from cruciferous plants, such as broccoli, Brussel sprouts or cabbages, confers a protective effect against AFB_1_-mediated genotoxicity in human hepatocytes [63]. Sulforaphane effectively induces hepatic total GST activity and attenuates hepatic AFB_1_-DNA adducts in AFB_1_-exposed rats [64,65]. In addition, the thyme and calendula extracts alone or in combination ameliorate aflatoxin-induced oxidative stress and genotoxicity mechanistically demonstrated the alterative expression of p53, bax and bcl2 gene expression [66]. Bhattacharya *et al.* [67] screened 26 plant phenolic flavonoids and found that the polyhydroxy flavonols, such as robinetin, quercetin, fisetin and morin, have a strong protective activity against the carcinogenic effects of AFB_1_.

However, the protective role of resveratrol against mycotoxicosis by other genotoxic mycotoxins is a controversial issue. Raghubee *et al.* [68] found that resveratrol, a polyphenol derived from red grape, blueberries, raspberries, mulberries, peanuts and itadori, ameliorates OTA-induced cellular oxidative stress in human embryonic kidneys. In addition, aflatoxin-induced AST, ALT and SOD were reduced by resveratrol supplementation in broiler birds [69]. However, Agamy *et al.* [70] compared the hepatoprotective effect of curcumin and resveratrol on aflatoxin-induced liver injury; curcumin, but not resveratrol, had a protective effect against AFB_1_-induced liver toxicity. Moreover, resveratrol has been shown to exert no protective effect against the cytotoxicity of mycotoxins, such as DON and OTA, in intestinal epithelial cells [71]. Therefore, more systematic and chronic effects of resveratrol on the mycotoxin-induced genotoxicity need to be assessed to determine the ultimate counteraction of the pharmacological components in the medicinal herbs and spices.

In addition to the effects of the medicinal natural components in herbs and spices on aflatoxin action, other mycotoxin-induced toxicities are also counteracted by these pharmacological elements. Yang *et al.* [72] reported that 6-gingerol, an active constituent of fresh ginger, has a strong protective property against the genotoxicity caused by patulin and that the antioxidant effect of 6-gingerol may play a critical role in reducing genotoxicity in hepatocytes. Several studies have reported the cytoprotective effect of epigallocatechin-3-gallate (EGCG), the most abundant polyphenol in green tea, against inflammatory responses caused by mycotoxins [73,74]. In particular, ribotoxic mycotoxins, such as DON and HT-2, exert diverse toxic effects on HT-29 cells by inducing oxidative stress, stimulating cyclooxygenase-2, enhancing caspase-3-activated apoptosis and stimulating the transcription of nuclear factor kappa B-mediated inflammatory genes. Lycopene has been considered as one of the most powerful antioxidants and is mainly found in tomatoes and other red fruits and vegetables, including red carrots, watermelons, gac and papayas. Lycopene also possesses protective activities against acute ZEA-triggered oxidative, inflammatory, endocrine and reproductive damage in mice [75,76].

**Table 2:** Effects of phytochemicals in medicinal herbs and spices on mycotoxicosis.

Types of inhibition	Herbs and Spices	Effects on mycotoxicosis	References
fInhibition of fungal growth	Ajowain	*A. flavus*, *A. parasiticus*	[33]
	Basil	*A. flavus*, *A. parasiticus*, *A. ochraceus*, *F. moniliforme*	[40]
	Cloves	*Aspergillus*, *Penicillium*	[35,36]
		*A. flavus*, *A. parasiticus*	[33]
	Clove oil	*A. flavus*, *A. parasiticus*	[33]
	Cinnamon	*Aspergillus*, *Penicillium*	[35,36]
		*A. flavus*, *A. parasiticus*, *A. ochraceus*, *F. moniliforme*	[40]
		*A. flavus*, *A. parasiticus*	[33]
	Chinese cassia	*Aspergillus*, *Penicillium*	[35]
	Coriander	*A. flavus*, *A. parasiticus*	[33]
	Kalonji	*A. flavus*, *A. parasiticus*	[33]
	Kalonji oil	*A. flavus*, *A. parasiticus*	[33]
	Marigold	*A. flavus*, *A. parasiticus*, *A. ochraceus*, *F. moniliforme*	[31]
	Neem oil	*A. flavus*, *A. parasiticus*	[33]
	Quyssum	*A. flavus*, *A. parasiticus*, *A. ochraceus*, *F. moniliforme*	[40]
	Spearmint	*A. flavus*, *A. parasiticus*, *A. ochraceus*, *F. moniliforme*	[40]
	Thyme	*Aspergillus*, *Penicillium*	[35]
		*A. flavus*, *A. parasiticus*, *A. ochraceus*, *A. fumigatus*, *Fusarium* spp*.*	[42,49]
	Thyme oil	*A. flavus*, *A. parasiticus*, *A. ochraceus*, *F. moniliforme*	[40]
	Turmeric	*A. flavus*, *A. parasiticus*	[33]
Inhibition of mycotoxin production	Anise	Sterigmatocystin, citrinin	[18]
	Black cumin	AFB, sterigmatocystin, citrinin	
	Black pepper	AF, sterigmatocystin	
	Peppermint	AFB_1_, citrinin	
	Cardamom	AFB_1_, sterigmatocystin, citrinin	
	Clove	AF, sterigmatocystin, citrinin	
	Cumin	AFB_1_, citrinin	
	Ginger	Sterigmatocystin	
	Marjoram	AFB_1_, citrinin	
	Sweet basil leaves	AFB_1_	[39]
Inhibition of mycotoxin actionn	Caffeic acid phenethyl ester	AFB_1_	[49,59]
		Normalization of γGT, ALP, GST and NO	
	Catechin	AFB_1_	[59]
		Attenuation of DNA adduct formation	
	Chitosan	AFB_1_	[50]
		Normalization of AST and ALT levels	
	Chlorogenic acid	AFB_1_	[59]
		Attenuation of DNA adduct formation	
	Turmeric	AFB_1_	[47,48]
		Normalization of LDH and ALT	
	Cyanidin	AFB_1_, OTA	[52,53,54,55]
		Normalization of ROS, protein and DNA synthesis, and apoptosis in HepG2 and Caco-2 cells	
	Diallyl sulfide	AFB_1_	[61]
		Reduction of DNA damage	
	Epigallocatechin-3-gallate	Deoxynivalenol, HT-2 toxin	[73,74]
		Suppression of inflammatory responses	
	Eugenol	AFB_1_	[59]
		Attenuation of DNA adduct formation	
	Fisetin	AFB_1_	[59,67]
		Prevention of carcinogenesis Attenuation of DNA adduct formation	
	Genistein	AFB_1_	[62]
		Reduction of mutagenesis	
	Indole-3-carbinol	AFB_1_	[77]
		Prevention of carcinogenesis in rat liver	
	Kaempferol	AFB_1_	[59]
		Attenuation of DNA adduct formation	
	Lycopene	AFB_1_, OTA ZEA	[56,57,58,75,76]
		Protection effect on oxidative, inflammatory, endocrine and reproductive damage in mice	
	Morin	AFB_1_	[59,67]
		Prevention of carcinogenesis Attenuation of DNA adduct formation	
	Naringin	AFB_1_	[59]
		Attenuation of DNA adduct formation	
	Quercetin	AFB_1_	[67]
		Prevention of carcinogenesis	
	Robinetin	AFB_1_	[67]
		Prevention of carcinogenesis	
	Sulforaphane	AFB_1_	[63,64,65]
		Induction of hepatic total GST activity. Attenuation of DNA adduct formation	
	Thyme oil	AFB_1_	[46,66]
		Excretion of AFs Normalization of AST, ALP and γGT Ameliorative effect on oxidative stress and genotoxicity	
	Vanillin	AFB_1_	[59]
		Attenuation of DNA adduct formation	
	Gingerol	Patulin	[37]
		Reduction of DNA damage in HepG2	

## 5. Conclusions

Although the occurrence and exposure of mycotoxins in most medicinal herbs and spices in various countries are inevitable, many of the bioactive components in these agricultural commodities have been known to regulate the fungal growth, mycotoxin production and their toxic actions in the plant and its herbivores, including human beings and domestic animals. These endogenous components are thus crucial attenuators by reducing the inevitable exposure and toxicities when taken in together with the contaminated mycotoxins. Since it is not easy to completely eliminate or prevent mycotoxin contamination during pre- and post-harvest stages, active strategies for reducing fungal growth and mycotoxin production are important for minimizing the exposure and toxicity to humans and animals. Although numerous methods for the elimination of mycotoxins using physical, chemical and biological strategies have been suggested, the safety of these methods and reducing agents still remain unclear. Since many components from medicinal herbs or spices affect the fungal growth or actions of mycotoxins, these antagonizing factors, considered safe and natural, have been studied to help alleviate mycotoxin exposure and toxicity. In addition to the regulatory actions of these endogenous components on fungal growth and mycotoxin production, these antagonizing components when mixed with mycotoxins would potentially reduce the risk of toxicity to exposed humans and animals. The compensatory actions of the endogenous beneficial components in terms of metabolism, distribution, excretion and final adverse actions against cells and tissues would be useful strategies to enable safe use of the medicinal herbs and spices unavoidably contaminated with mycotoxins at levels under the regulatory limits. However, since some natural endogenous components could enhance the toxicity of mycotoxins via metabolic activation or retarded secretion by complex formation with mycotoxins, extensive investigations into these interactions is warranted for the sound toxicological assessment of herbal medicines and spices for human and animal use in the future. In summary, the biological interaction between mold, mycotoxin and herbal components might be an important strategy for overcoming the worldwide occurrence of mycotoxin contamination, including that in medicinal herbs and spices. This review presented the safest, cost-efficient and most natural strategies for better risk assessment of mycotoxin-contaminated medicinal herbs and spices in the environment and in the food chain.
